# Impact of high-energy photon irradiation on early-stage dissolution of EAF slag and brownmillerite

**DOI:** 10.1038/s41598-025-22779-5

**Published:** 2025-11-05

**Authors:** Recep Kurtulus, Kalle Inget, Cansu Kurtulus, Mahtab Akbarzadeh Khoei, Marco Cantaluppi, Sakari S. Karhula, Juha Nikkinen, Otto Mankinen, Juho Yliniemi

**Affiliations:** 1 Fiber and Particle Engineering Research Unit, University of Oulu, Oulu, PO Box 4300, 90014 Finland; 2 Department of Materials Science and Engineering, Faculty of Engineering, Afyon Kocatepe University, Afyonkarahisar, Turkey; 3 Department of Oncology and Radiotherapy, Oulu University Hospital, Oulu, Finland; 4 Medical Research Center, Oulu, Finland; 5 Department of Chemical Engineering, Faculty of Engineering, Afyon Kocatepe University, Afyonkarahisar, Turkey; 6 Research Unit of Health Science and Technology, University of Oulu, Oulu, Finland; 7 NMR Research Unit, University of Oulu, Oulu, Finland

**Keywords:** EAF slag, Dissolution, High energy photon, Brownmillerite, Waste utilization, Green chemistry, Inorganic chemistry, Materials chemistry, Applied physics

## Abstract

The influence of external conditions on the dissolution of minerals within inorganic sidestreams, such as steel slags, is a critical factor when considering their utilization pathways. This study addresses the aqueous dissolution characteristics of electric arc furnace slag (EAFS) and one of its main crystal phases – brownmillerite (BM), and delves into the impact of high energy photon irradiation (HEPI). The untreated forms of EAFS and BM were exposed to HEPIs using Cs-137 isotope (0.662 MeV, 250 Gy) and medical linear accelerator (10 MeV, 52 kGy) for 72 h and 16 h, respectively. The impact of HEPIs on dissolution was quantified based on batch dissolution experiments in water under ambient conditions with a solid-to-liquid ratio of 1:100 g/mL. Afterward, a systematic characterization series is conducted to understand structural changes, surface alteration, and solution chemistry in EAFS and BM samples. XRD and FTIR analysis reveal that exposure to different HEPIs caused almost no structural changes in both powders. In contrast, SEM analysis shows that HEPIs led to prominent microcracks on BM’s surface, with slight variations on EAFS. The extent of dissolution for Al and Ca ranges from 5% to 10% and 3% to 5% over time for the untreated BM, respectively, and these values are, at least, doubled when HEPIs is applied. For the case of EAFS, similar enhancements via HEPIs are achieved compared to its untreated form, but with higher Ca and Al extents. The enhancement in dissolution is associated with the micro-cracks, as evidenced by SEM analysis. To conclude, HEPIs can affect the early-stage dissolution properties of EAFS and BM to a certain degree, and more elements can be released if a high-energy photon dose is applied.

## Introduction

The continual demand for iron and steel materials across diverse applications has significantly increased global production capacities. Beginning at 200 Mt/year in the 1950s, iron and steel production have lately reached amounts tenfold greater^1,2^. Notwithstanding this, the projected yearly quantities are expected to range from 2500 to 3000 Mt/year by the 2050s^3^. Despite its popularity and prevalence, the widespread use of iron and steel undoubtedly poses significant environmental dangers due to the extensive consumption of primary raw materials and fossil fuels during production, and the generation of leftover slags as industrial byproducts^4^. Specifically, each tonne of manufactured iron and steel generates 150 to 200 kg of slag, depending on the production conditions^5^. Considering the annual production capacities, the formation of iron and steel slags cannot be underestimated since they are landfilled in nature without any benefit but acting as ecological pollutants^6,7^. Consequently, it is imperative to promote waste management strategies that facilitate responsible and sustainable utilization of iron and steel slags.

Iron and steel materials are mainly manufactured by blast furnace (BF) and electric arc furnace (EAF) processing techniques. The former utilizes iron ore and coke, together with slag formers such as calcite or dolomite, and subsequently processes the mixture at high temperatures ranging from 1800 to 2000 °C^8^. The latter, conversely, employs scrap iron and steel derived from end-of-life products (e.g., vehicles or home appliances), production wastes (e.g., rejected parts or off-cuts), and outdated machineries (e.g., ships or factory equipment), besides utilizing slag formers^9^. Currently, the BF process is generally preferred over the EAF for iron and steel production, representing 75% of total output^10^. Nonetheless, the key drawbacks of the BF process, including intense natural resource use and high carbon dioxide emissions, have driven the industry to seek greener solutions. In this sense, the iron and steel sector has firmly concentrated on a green transition to achieve the carbon-neutral objectives of the Green Deal^11–13^. Hence, many companies have been considering or acting shifting production methods from BF to EAF combined with hydrogen energy to pursue environmentally-friendly manufacturing practices.

While recognizing that the green transition in iron and steel production would enhance sustainability through the EAF technology, a significant challenge persists in utilization of EAF slag (EAFS). Largest volumes of EAFS could be utilized in applications such as concrete^14,15^ or in low-value road construction applications^16,17^. In concrete, for example, EAFS could potentially replace Portland cement or fine or coarse aggregates. The low cementitious reactivity of EAFS often prevents its effective use as cement replacement, but the high density of EAFS could give additional value in its use as aggregate replacement, for instance, in concrete that needs nuclear radiation shielding property. This is because, the high density and specific elemental composition of EAFS, especially its enrichment in iron, calcium, and trace heavy metal oxides, make it a promising candidate for radiation shielding applications^18,19^. Given the inadequate waste management of EAFS, further research and development efforts are crucial for effectively utilizing current and future occurrences. Most importantly, the circular economy model must be formulated for the iron and steel sector, incorporating EAFS with added value. As a result, researchers should thoroughly examine the application of EAFS and their possible challenges.

For the broader usability of EAFS, structural complexity, one of the most compelling limitations, has become a primary focus of the research community. In contrast to BF slag, which mainly consists of an amorphous phase and minor minerals such as merwinite or akermanite^20,21^, EAFS exhibits a mostly crystalline structure and contains hardly any amorphous phases. The highly crystalline structure of EAFS is composed of various phase assemblages, including larnite, gehlenite, brownmillerite, merwinite, bredigite, spinel, RO-phase, and wuestite^22,23^. These mineral phases and their proportions are affected by variations based on the chemical composition of iron and steel production in addition to the final cooling rate of the slag. The structural complexity, presence of stable mineral phases, and lack of amorphism in EAFS limit its wide implementation in supplementary cementitious materials, alkali-activated materials, concrete aggregate, valuable metal recovery, carbon capture, mineralization, and nuclear waste immobilization applications. The characteristics of early-stage dissolution directly influence the larger use of EAFS within the given applications. Therefore, a comprehensive understanding of the dissolution behavior of EAFS under varying conditions (e.g., pH or time) is the key factor for future applicabilities.

In the context of mineral dissolution, several critical parameters influence the final breakdown properties, such as mineralogy (e.g., crystal structure, impurities, or defects), ambient conditions (e.g., solution pH, temperature, or pressure), and external treatments (e.g., irradiation, time, or flow)^24,25^. The structural complexity of EAFS is a potential barrier that may be addressed through modifications in ambient conditions and external treatments. Earlier studies in the literature have sought to reduce the particle size of EAFS by physicomechanical and mechanochemical methods to increase the specific surface area, and thereby improve particle reactivity^26,27^. Additionally, the majority employed various pH solutions, ranging from highly acidic to basic levels, and temperature conditions to chemically and thermally dissolve the EAFS particles^28–30^. As a result of these efforts, the researchers have acquired insights into the dissolution process of EAFS, especially about the behavior of several mineral phases, structural alterations, and surface modifications. According to these reports, the iron-bearing phases, such as wuestite or RO-phase, can be dissolved in acidic conditions at somewhat elevated temperatures^31,32^. Conversely, silicate-containing phases such as larnite or gehlenite are soluble under alkaline conditions at increased temperatures^33^. In addition, calcium-containing phases such as calcite or mayenite can dissolve under neutral conditions with moderate temperatures^34,35^. Although acquiring essential knowledge to dissolve EAFS, the process still requires additional enhancements. Because the extensive use of chemicals to attain highly acidic or basic conditions results in environmental damage, safety risks at work, and difficulties in industrialization. Moreover, the requirement for elevated temperature conditions is a principal factor constraining implementation within numerous application areas. Yet more, the element releases are still inadequate in promoting the extensive utilization of EAFS. With these in mind, the scientific community seeks to address the issue of EAFS by minimizing chemical usage, achieving green hydrometallurgy, modifying particle surfaces, damaging crystal structures, and improving element releases^36^. Therefore, the researchers aim to enhance early-stage dissolution properties to broaden the existing knowledge in possible application areas.

In addition to mineralogy and ambient condition factors, further insights are necessary to explore the effects of external treatments, especially irradiation techniques (IrrTechs). IrrTechs can modify the crystal structure of a mineral, potentially resulting in amorphism or structural modification, depending upon the energy level applied and the crystal phase assemblage^37–39^. Besides structural modifications, IrrTechs may modify the particle surface to induce thermal stress from irradiation energy, leading to the propagation of microcracks of varying lengths and widths^40,41^. These microcracks allow a gradual increase in particle surface area relative to the untreated form, facilitating more significant contact between liquids and solids, which may enhance dissolution behavior. That said, IrrTechs may be applied to the solid forms of the mineral or during the dissolution process. As a result, IrrTechs possesses a potentiality in terms of significant external treatment for promoting early-stage dissolution.

The existing literature includes studies examining the effects of IrrTechs on the improvement of dissolution properties of minerals, as well as industrial sidestreams. Dong et al.^42^ investigated ultrasound irradiation use to increase element extraction from different industrial sidestreams, such as blast furnace and stainless steel slags. Similarly, John et al.^43^ examined the leaching of lead elements from accumulated metallurgical waste through ultrasound irradiation. On the other hand, Attah et al.^44^ studied the application of microwave irradiation to recover vanadium elements from steel converter slag as another IrrTechs. Likewise, Laubertova et al.^45^ explored the impact of microwave irradiation on the leaching properties of EAF dusts. In addition to low-energy IrrTechs (i.e. ultrasound or microwave), Hsiao et al.^46^ researched the impact of neutron irradiation on calcite and dolomite minerals as high-energy IrrTechs, particularly focusing on changes in atomic structure and chemical durability. Besides that, Dukes et al.^47^ assessed the effects of Ar^+^ ion bombardment on olivine minerals and conducted an aqueous dissolution test to analyze surface atomic changes and their impact on dissolution behavior. Within the context of other IrrTechs, Plötze et al.^48^ examined gamma-rays’ effects on clay minerals, focusing on their physicochemical properties to enhance understanding of potential application areas. Gawel et al.^38^ evaluated the structural changes in olivine minerals by applying different gamma-ray doses to determine their potential for nuclear radiation applications. Gore et al.^49^ researched the leachability of beach sand, basaltic sediments, and mineralized base metal ores, as well as evaluate their sterilization efficiency following gamma irradiation. Yeoh et al.^50^ considered the long-term gamma-ray exposure of alkali-activated pastes composed of blast furnace slag and fly ash to understand the alterations in their physical, chemical, structural, and mechanical properties to utilize industrial sidestreams effectively. As can be seen, these studies have provided fundamental insights into the effects of energy, dose, and time and their implications for a mineral’s structure and surface characteristics. Nevertheless, there remains a significant gap in the literature, particularly regarding understanding industrial sidestreams, specifically steel slags (e.g., EAFS), under various IrrTechs. The possible influence of high energy photon irradation (HEPI) on early-stage dissolution of minerals will influence EAFS utilization potential as cement and aggregate replacement material in concrete and in earth construction applications, because in all of those the chemical interactions are largely governed by surface dissolution-precipitation reactions between the surrounding matrix and the dissolving mineral. Therefore, this study used HEPI with Cs-137 isotope and linear acceleator to induce structural and surface modifications in EAFS and one of its main crystal phases, brownmillerite, and then conducted thorough characterization analyses. Both untreated and high energy photon-irradiated sample series were investigated with batch dissolution experiments in water at ambient conditions.

## Materials & methods

### Materials

The electric arc furnace slag (EAFS) used in this investigation was the same as in^51^. EAFS was milled to median size of less than 30 microns to facilitate sufficient contact area between solid and liquid phases. Table 1 presents the oxide contents based on the chemical composition determined using the X-ray fluorescence (XRF) technique. Using X-ray diffraction (XRD) technique (9 kW Rigaku Smartlab instrument), the mineralogical composition was measured from 10 to 130 2-theta degrees. Phase identifications were done using the PDXL2 software, specifically with PDF- 4 + 2023 database. In addition, details relating to the preparation of synthetic brownmillerite (BM) mineral are available in our earlier study^51^.

**Table 1:** Chemical composition of electric Arc furnace slag (in wt%)^47,48^.

CaO	SiO_2_	Al_2_O_3_	Fe_2_O_3_	MgO	*P*_2_O_5_	TiO_2_	SO_3_	Cr_2_O_3_
38.0	14.8	7.6	27.3	8.0	1.2	0.7	0.6	0.07

### High energy photon irradiation techniques

#### On the study for synthetic brownmillerite

**Fig. 1:**
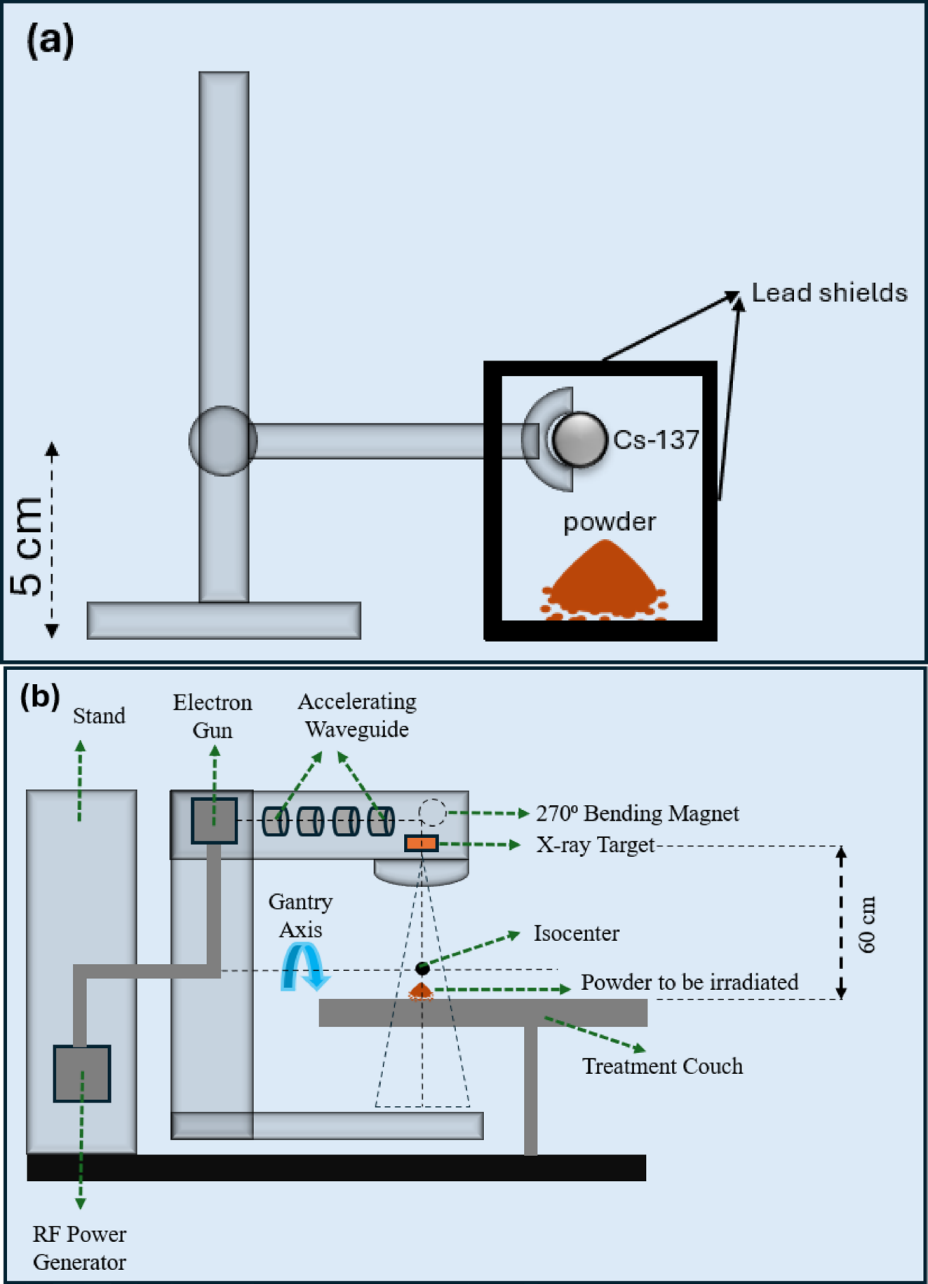
The schematic representation of experimental setups of the BM and EAFS irradiations. Powder represents BM or EAFS as 5 and 10 g, respectively. Exposure times are 72 and 16 h with Cs-137 (**a**) and linear accelarator (**b**).

The effect of high energy photon irradiation (HEPI) on the synthetic brownmillerite (BM) and electric arc furnace slag (EAFS) was performed using two different methods to produce high energy photons: Cs-137 isotope (0.662 MeV, total dose: 〜250 Gy) and medical linear accelerator (10 MeV, total dose: 〜52 kGy). Figure 1 demonstrates the HEPI schematic setups of the synthetic BM and EAFS, respectively. The experimental setup for the Cs-137 source (hereafter called UO) was done in the laboratory conditions (University of Oulu), in such a way that starting with the corresponding powders (i.e., synthetic BM and EAFS) followed by their exposure to UO for 72 h and finally obtainment of the high energy photon-irradiated powders. The distance from the source to the powders was around 5 cm in height (see Fig. 1a). For the case of the medical linear accelerator (hereafter called UOH), a similar procedure was followed, but the high-energy photons originated from a TrueBEAM (Varian A Siemens Healthineers Company) radiotherapy instrument located at the Oulu University Hospital. The corresponding powders were exposed for total of 16 h irradiation with linear accelerator. The irradiation was conducted with 10 MeV photon energy, without flattening filter to achieve dose rate of 2400 monitor units per minute (MU/min). In our measurement setup, this corresponds to approximate exposure rate of 61.2 Gy/min in the powder. Due to the instrument’s configuration, the distance was adjusted to 60 cm on top of those BM and EAFS powders (see Fig. 1b). As a result, four different high-energy photon irradiated BM and EAFS powders, coded as *Irr-UO-BM*, *Irr-UOH-BM*, *Irr-UO-EAFS*, and *Irr-UOH-EAFS*, were successfully obtained.

### Batch dissolution experiments

Batch dissolution experiments were conducted for untreated reference and high energy photon irradiated powders. The ratio of powder to deionized water was 1:100 g/mL, and the necessary amounts were mixed in a glass beaker. Under ambient conditions, a magnetic stirrer (Velp Scientifica Arex Digital Pro) agitated the solutions (BM and EAFS series) at 330 rpm for 120 min with a step size of 30 min for each sample. From now on, the sample codes will be said as, for example, *Irr-UO-BM-30* to *Irr-UO-BM-120* to signify the dissolution test intervals.

### Characterizations

Once each dissolution time interval was completed, vacuum-filtration (VWR, Ø- 75 mm filter paper with a 5–13 μm particle retention) was applied to filter the corresponding solutions. Afterward, the aliquots were collected using a syringe fitted with a 0.22 μm filter, followed by acidification to pH < 2 with 0.1 M HNO_3_ solution. Each sample series was then kept in the fridge (at 4℃) prior to the analysis. Besides that, for each sample series, the solid residues left over the filter paper from vacuum filtration were washed with milli-Q water, dried in the oven at 105 °C for 24 h, and finally kept in a desiccator until characterization analyses.

The elements Ca, Si, Al, Fe, Mg, and Ti concentrations were searched using Inductively Coupled Plasma Optical Emission Spectroscopy (ICP-OES) analysis in compliance with EN ISO 11,885. It is essential to note that after the dissolving experiments, a few randomly chosen solutions were collected three times and subjected to ICP-OES analysis. The findings demonstrated that the error percent fluctuates between ≤ ± 10%. Consequently, the dissolution concentration plots’ error bars show ± 10% in accordance.

After obtaining ICP-OES results, the element concentrations were used to calculate the dissolution extent percents (*DE%*) by applying Eq. 1^52^. One should consider that precipitation or adsorption phenomena may concurrently occur along with the dissolution process, strongly influencing *DE%*. For this reason, the only aim for calculating DE% is to comprehend the variations in dissolution as a function of time.

1 $$\:DE\:\left(\%\right)=\:\frac{{C}_{i}\:.\:V}{m\:.\:{x}_{i}}.\:100$$

Here, *C*_*i*_, *V*, *m*_*i*_, and *x*_*i*_ signify the concentration of element-*i* (mg/L), volume of the solution (L), mass of the solid in solution (g), and mass fraction of element-*i* in solid determined by XRF analysis, respectively.

Following the completion of the corresponding dissolution times, the solution pH was measured using the inoLab pH7110 instrument, which had an error margin lower than 0.1 pH units. Prior to measurements, pH electrode was calibrated using buffer solutions, namely pH 7 and 10 (VWR Chemicals).

Fourier transform infrared (FTIR) technique was applied to the dried solid residue samples to understand the alterations in the vibrational modes. The spectral data were acquired between 4000 and 400 cm^− 1^ via a Bruker Vertex v80 device with a nitrogen-cooled MCT detector, operating in absorbance mode under ambient air conditions.

X-ray photoelectron spectroscopy (XPS) was used to determine the samples’ surface element concentration and chemical state. The spectral data were collected using a Thermo Fisher Scientific ESCALAB 250 Xi instrument having an X-ray source (Al-Kα, 1486 eV energy). The gathered data were assessed using Avantage Software upon calibrating adventitious carbon (C 1s, 284.8 eV).

Brunauer-Emmett-Teller (BET) method using the ASAP 2020 Micrometrics device was performed to determine the specific surface area of the related samples.

Scanning electron microscopy (SEM) via a Zeiss Sigma FESEM device under 15 kV voltage was employed to observe the surface morphology of the related samples. Prior to the analysis, the particles were put on a carbon plate and then coated using platinum to prevent the charge-up effect.

## Results

### Structural investigation

**Fig. 2:**
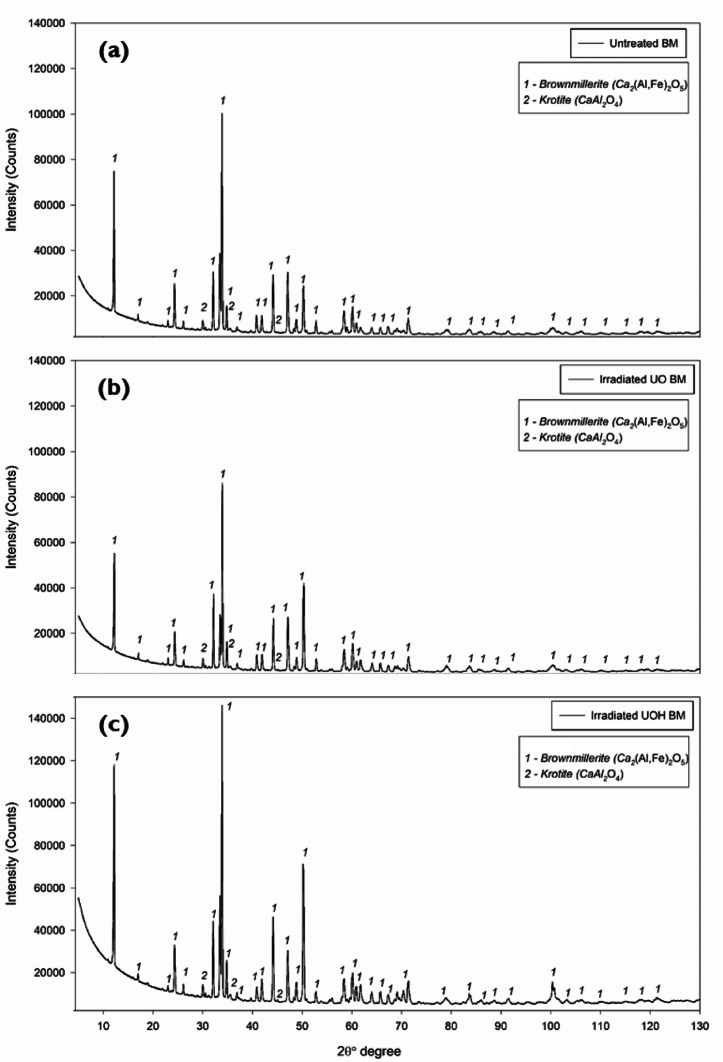
XRD patterns of the untreated^47^ (**a**), Cs137 irradiated (**b**), and 10 MeV irradiated (**c**) synthetic brownmillerite.

The X-ray diffraction (XRD) patterns of the untreated BM are presented in Fig. 2a. The prominent peaks identified are brownmillerite (PDF #04-015-6868) and krotite (PDF #04-013-0779), of which the former has 93.5 wt% and the latter has 6.5 wt% in quantity, according to the Rietveld quantification analysis. The synthesized BM shows a typical XRD pattern compared to the literature studies^53,54^. When HEPI procedures are applied to the untreated BM, their patterns are shown in Fig. 2b and c, respectively. In each case, the BM structure has almost similar crystallography, irrespective of the applied procedures. The exposure dose and duration did not result in significant alterations. Therefore, it can be said that with irradiation total dosage of 52 kGy and 250 Gy and duration of 16 h and 72 h, there is no structural damage on brownmillerite and krotite.

**Fig. 3:**
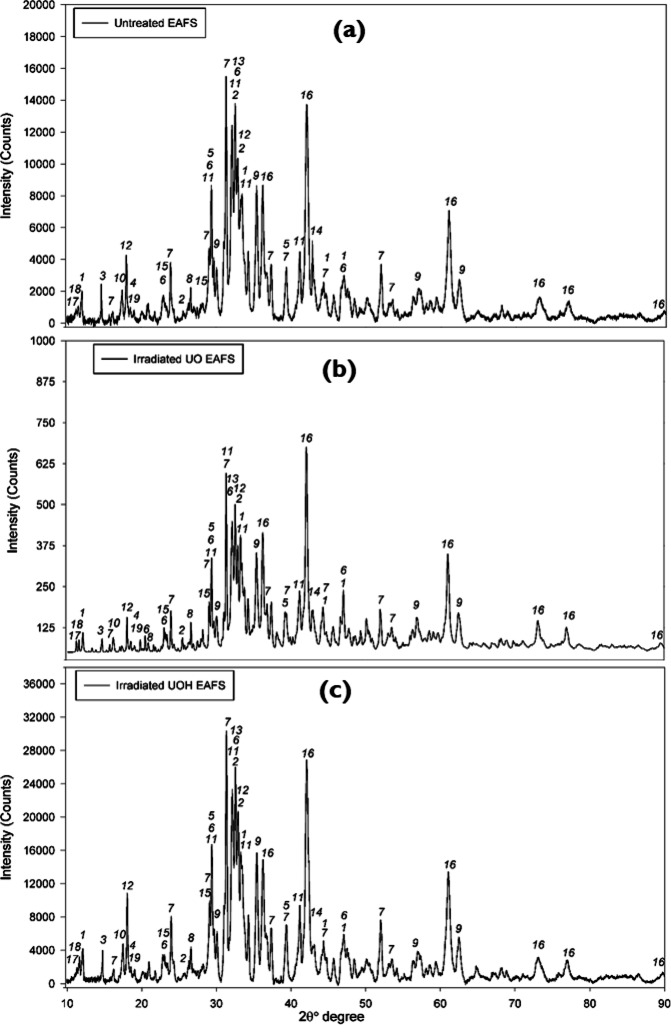
XRD patterns of the untreated^48^ (**a**), Cs137 irradiated (**b**), and 10 MeV irradiated (**c**) EAF slag.

To evaluate the crystal structure of the untreated electric arc furnace slag (EAFS), Fig. 3a displays the XRD patterns. The identified peaks, on the one hand, can be seen in Table 2. EAFS has complex structure with multiple mineral phases. The prominent crystal phases are brownmillerite (#1), bredigite (#2), gehlenite (#7), larnite (#11), merwinite (#13), and RO-phase (#16). These phases can also be found in the other literature studies dealing with EAFS^55,56^. After applying two different HEPI procedures, similar to the untreated BM, the XRD patterns are illustrated in Fig. 3b and c, respectively. When the patterns are examined in detail, one can deduce that almost no crystallographic alterations existed after HEPI procedures. That means the applied total doses and durations did not affect the crystal structure of EAFS, like in the case of the untreated BM. These outcomes indicate that higher total doses or longer duration would be needed to damage the crystal structures of EAFS.

**Table 2:** The identified crystal phases and their related numbers.

Number	Phase	Formula	PDF number
1	Brownmillerite	Ca_2_(Al, Fe^3+^)_2_O_5_	30–0226
2	Bredigite	Ca_7_Mg(SiO_4_)_4_	36–0399
3	Bassanite	Ca(SO_4_) 0.5H_2_O	41–0224
6	Brucite	Mg(OH)_2_	44-1482
5	Calcite	CaCO_3_	37-1492
6	Calcio olivine	ϒ-Ca_2_SiO_4_	49-1672
7	Gehlenite	Ca_2_Al[AlSiO_7_]	35–0755
8	Quartz	SiO_2_	46-1045
9	Spinel	MgAl_2_O_4_	33–0853
10	Hydrogarnet	Ca_3_Al_2_(OH)_12_	24–0217
11	Larnite	ß-Ca_2_SiO_4_	33–0302
12	Mayenite	Ca_12_Al_14_O_33_	09-0413
13	Merwinite	Ca_3_Mg(SiO_4_)_2_	35–0591
14	Periclase	MgO	45–0946
15	Rankinite	Ca_3_Si_2_O_7_	70-1138
16	RO-phase	(Ca, Mg, Fe, Mn)O	06-0615
17	Sjoegrenite	Mg_6_Fe_2_(CO_3_)(OH)_16_ 4H_2_O	74-1513
18	Hydrotalcite	Mg_6_Al_2_(CO_3_)(OH)_16_ 4H_2_O	89–0460
19	Nordstrandite	Al(OH)_3_	24 − 0006

### Dissolution characteristics

**Fig. 4:**
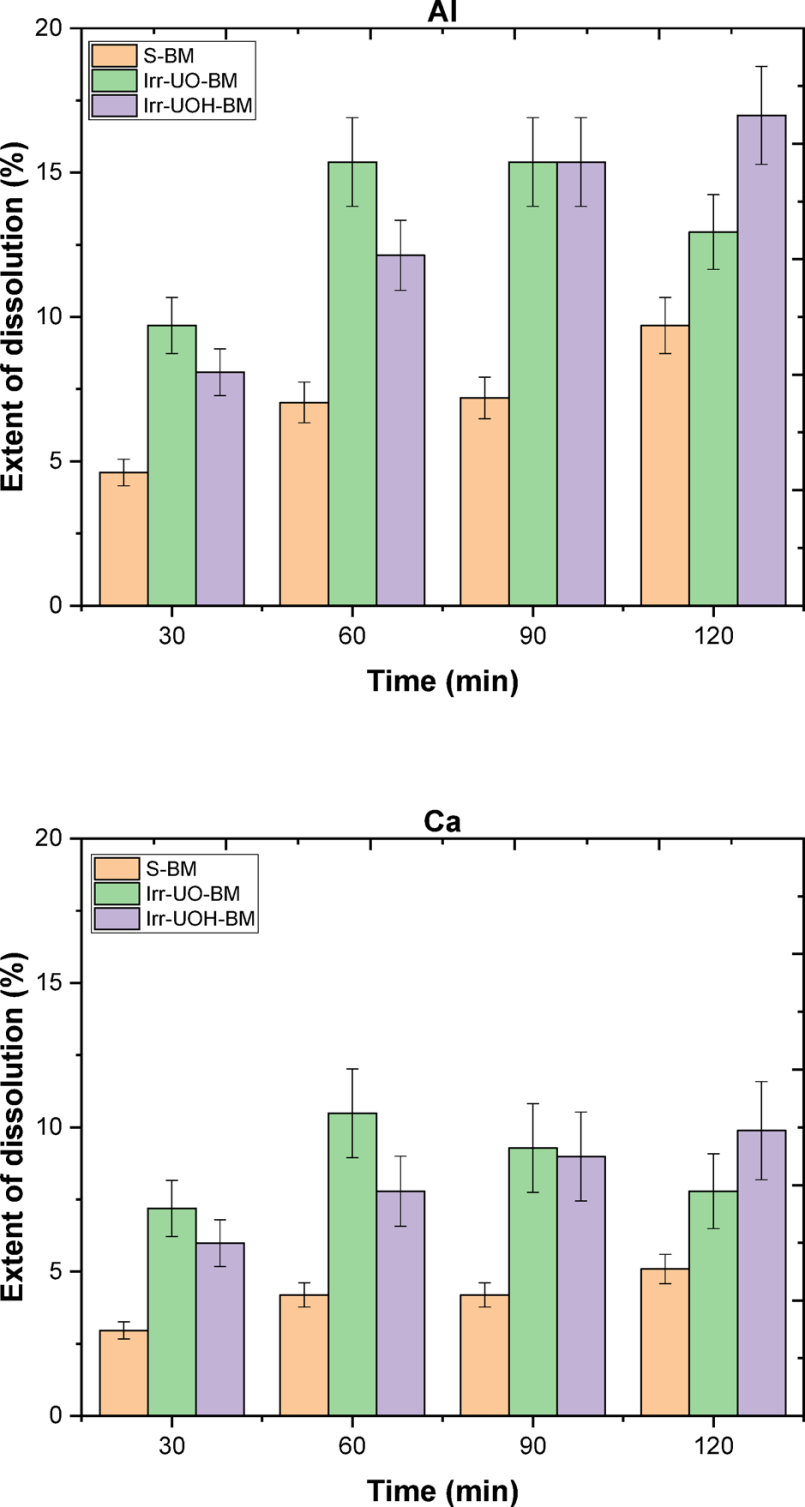
Extent of dissolution for the elements in the untreated and high energy photon-irradiated brownmillerite.

**Fig. 5:**
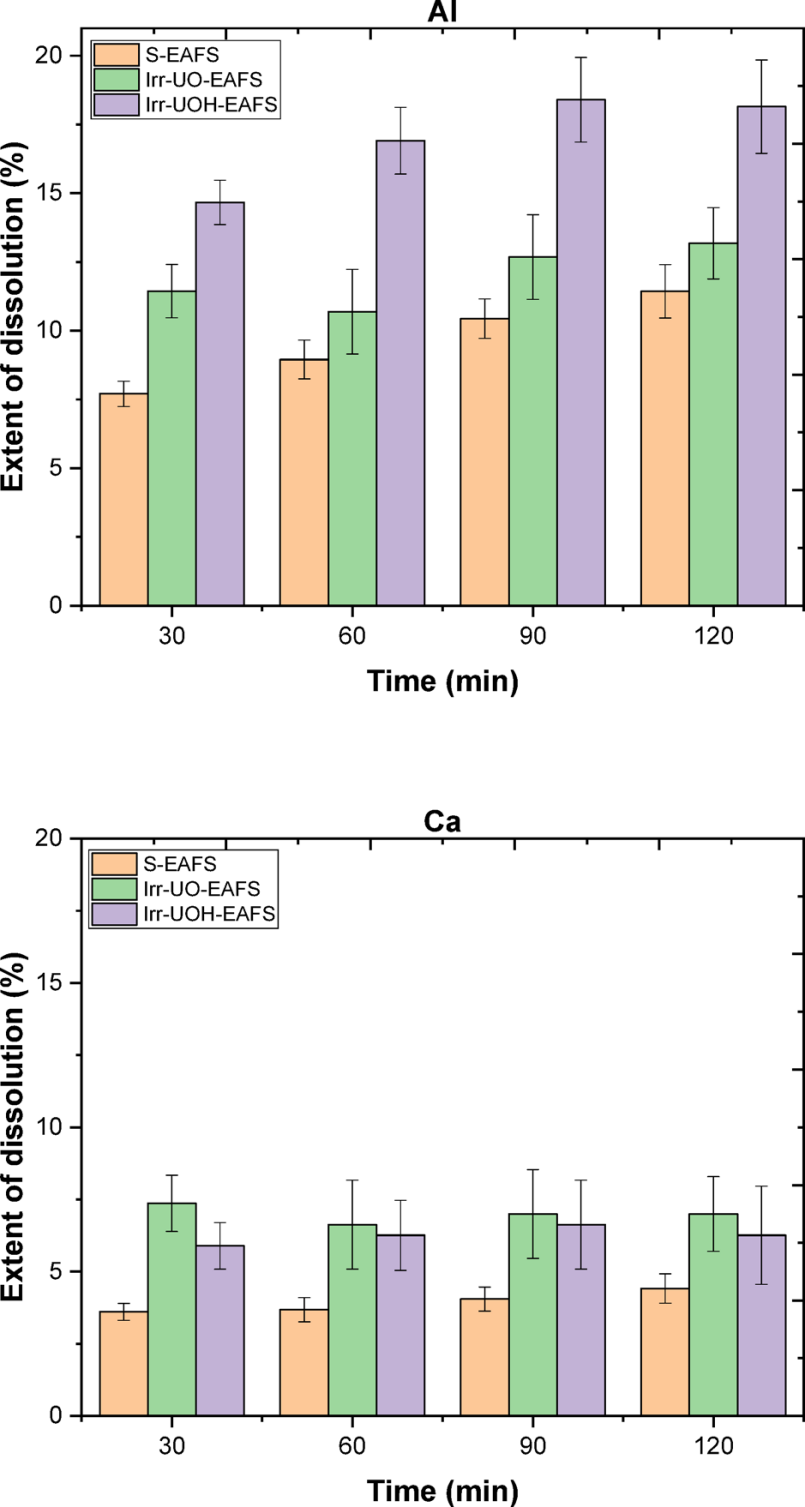
Extent of dissolution for the elements in the untreated and high energy photon-irradiated electric arc furnace slag.

The dissolution characteristics of synthetic brownmillerite (BM) and electric arc furnace slag (EAFS) have been examined utilizing the dissolution extent (*DE*%), as illustrated in Figs. 4 and 5, respectively. In the plots, codes *S-X* (*X*: BM or EAFS) reflect the untreated forms of the samples dissolved under stirring conditions, and codes *Irr-UO-X and Irr-UOH-X* indicate high energy photon irradiated samples. Following that, Fig. 4 illustrates the releases of Al and Ca, respectively. Since other elements, such as Si, Fe, Mg, or Ti, were not found in the detected ranges, the focus was only canalized toward Al and Ca in both sample series. In the case of Al release, it can be stated that both HEPI procedures enhanced the *DE*% relative to the reference sample series (i.e., S-BM). The *DE*% for the S-BM series fluctuates between 5% and 10% over time; however, these percentages are, at least, doubled when HEPI techniques are employed. The Irr-UO-BM and Irr-UOH-BM series exhibited variations ranging from 10% to 16% and 8% to 17%, respectively. Conversely, the release of Ca is lower than that of Al, varying between 3% and 5% for the S-BM series. Analogous to the impacts of gamma irradiation processes in Al, the release of Ca also doubled by the implemented procedures. The highest *DE*% is attained using two methods, which differ in their designated times. Besides evaluating both elements, one may additionally analyze the impact of various HEPI techniques. The first impact of the dissolution may be perceived as significant, particularly within the first 60 min for both aspects, with subsequent time allocation indicating an improvement in this condition. As a result of dissolution period, irradiation from the 10 MeV photons provides higher element releases than the Cs-137 source. The results may be attributed to the variation in photon energy levels and overall applied total doses, preferring the higher dose from UOH irradiation. In conclusion, both HEPI methods may exhibit increased element releases in the synthetic BM mineral.

In the context of the dissolution of the EAFS sample series, Fig. 5 illustrates the *DE*% for Al and Ca in that sequence (no Si, Fe, Mg, or Ti, were not found in the detected ranges). Applying the reference stirring approach (see S-EAFS) yields a *DE*% of Al between 7% and 10% as a function of time, while the *DE*% of Ca is comparatively lower, around 4% over time. Upon subjecting EAFS to HEPI techniques, the *DE*% of Al varies from 11% to 13% for UO samples and from 15% to 18% for UOH samples. The most significant alteration has been noted within the initial 60 min for both procedures, exhibiting a marginal increase after that. It is essential to note that the 10 MeV irradiated sample (UOH) facilitates a larger *DE*% than the Cs-137 source (UO) due to the former providing greater total doses. Conversely, the *DE*% of Ca rises when both HEPI methods are applied; nonetheless, it may not result in significant changes in *DE*%. The UO sample series has minor discrepancies from the UOH series; however, this may not be accurate due to the proximity of the error bars. Furthermore, the deficiency of *DE*% in Ca may be assessed by examining the potential precipitation and/or adsorption of Ca, such as Ca(OH)_2_. In conclusion, HEPI from the UOH source can facilitate more element releases than the Cs-137 source. The results may be associated with variations in photon energy levels and the total doses, indicating the preferred higher dose from UOH irradiation. Consequently, both HEPI techniques may yield increased element releases in EAFS.

In addition to dissolution extents, pH values were recorded for BM and EAFS at designated intervals. For BM, initial readings (〜2 min) were 11.0, 11.0, and 11.1 for the untreated, UO, and UOH samples, increasing to 11.8, 12.0, and 12.3 by the end of the test. For EAFS, initial values (〜2 min) were 11.9, 12.0, and 12.0, rising to 12.1, 12.3, and 12.6, respectively. These trends indicate that dissolution of Ca- and Al-bearing phases probably increased alkalinity, with irradiated samples consistently showing higher pH than their untreated counterparts, as also observed in our previous publications on Ca-rich phases under similar conditions^51,57^.

### FTIR measurements

**Fig. 6:**
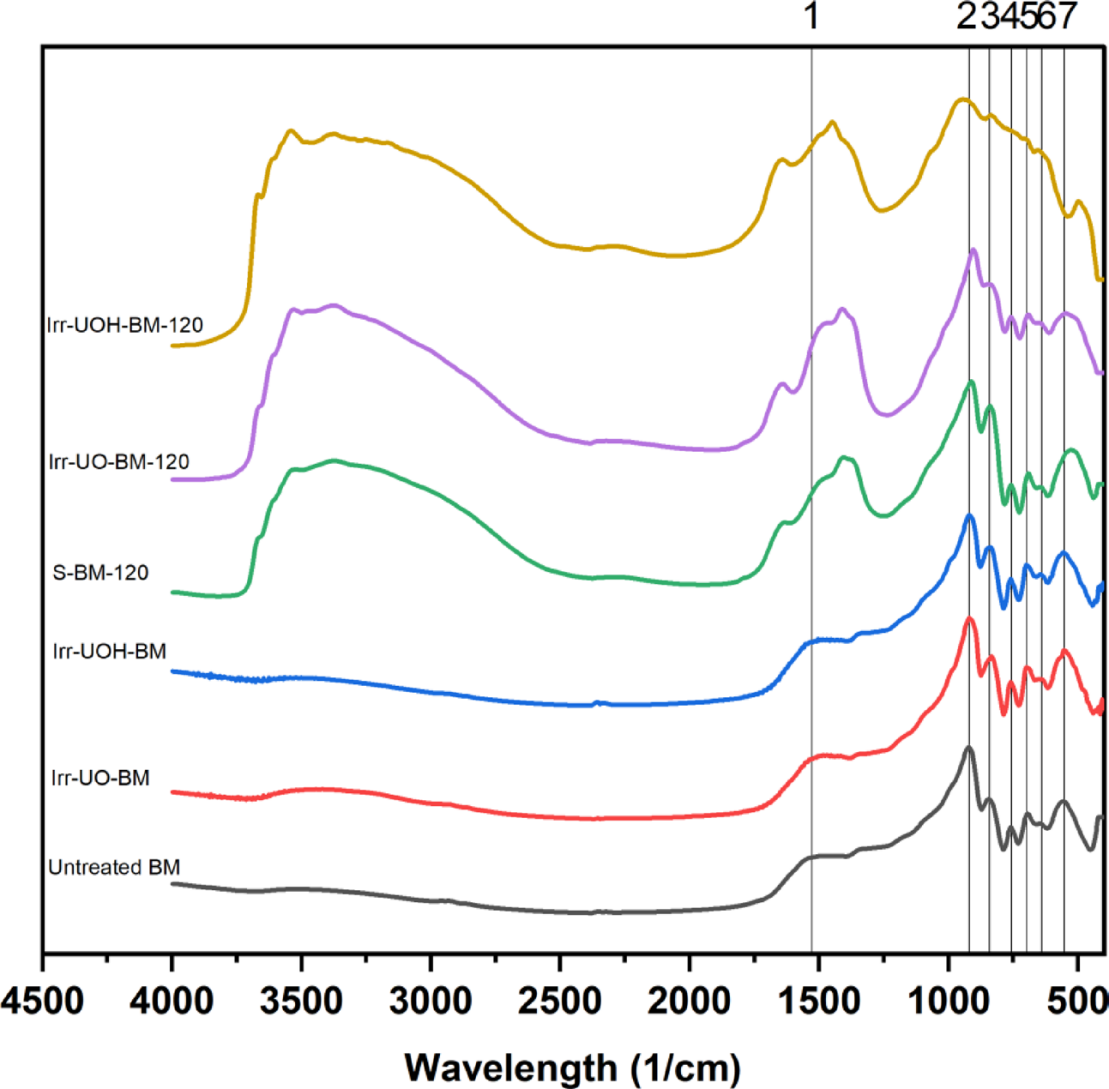
FTIR spectra of the synthetic brownmillerite sample series.

The FTIR technique was utilized on the corresponding sample series to analyze the vibrational modes before and following HEPI and the dissolution process. Figure 6 illustrates the spectra associated with the synthetic brownmillerite (BM) mineral. The left-hand side plots depict the untreated form at the top, with the high energy photon-irradiated samples, specifically the UO and UOH series, illustrated in the middle and bottom, respectively. The figures displayed on the right represent the spectra obtained after the 120-minute dissolution test. Furthermore, the dashed lines indicate the identified significant peaks from #1 to #7.

Based on the graph details, observations regarding the vibrational modes in the untreated BM sample can be made. The peak at approximately 1470 cm^− 1^ (#1) is associated with the formation of C-O vibrations due to atmospheric carbon dioxide^58^. In the fingerprint region, additional characteristic peaks are visible, such as the one observed near 920 cm^− 1^ (#2), which is linked to the stretching units of Fe-O-Fe bonds. A low-intensity peak seen at around 845 cm^− 1^ (#3) is likely due to the stretching vibrations of Al-O bonds^59^. Additional peaks identified at 755 cm^− 1^ (#4) and 695 cm^− 1^ (#5) might be associated with the vibrations of Ca-O bonds^60^, whereas subsequent peaks at 645 cm^− 1^ (#6) and 555 cm^− 1^ (#7) may correspond to the stretching modes of Fe-O bonds^61,62^. After identifying the bonds related to the untreated BM, attention can be directed towards the high energy photon irradiated samples. Irr-UO-BM and Irr-UOH-BM exhibited no significant changes in the vibrational units, with only minor alterations in peak positions noticed. The applied HEPI procedures did not change the bonds’ stretching and bending units.

The dissolution of untreated and high energy photon irradiated BM powders led to notable alterations in the vibrational modes, as illustrated on the right side. The primary distinction is observed in the formation of O-H vibration units (dashed rectangular), which range from 3600 to 3000 cm^− 1^ and exhibit a broader profile^14^. This situation may suggest surface modification due to hydroxyl attack and the dissolution process. A new peak at approximately 1635 cm^− 1^ appears, potentially associated with the formation of H-O-H vibrational modes^63^. Another notable change is the increasing peak intensity of C-O observed around 1450 cm^− 1^, indicating an enhancement of surface carbonation (e.g., CaCO_3_)^64^. Although the peak positions, such as #2 or #3, remained unchanged after the dissolution test, the associated peak intensities vary, especially those observed in the Irr-UOH-BM-120 sample. The results indicate that the element releases from the corresponding sample series significantly alter the vibrational units, particularly in the UOH series, compared to the UO and S samples. high energy photon irradiated samples exhibit comparable vibrational units to the untreated BM mineral; however, the dissolution process induces prominent alterations in the stretching and bending vibrations of the associated bonds, attributable to changes in element releases.

**Fig. 7:**
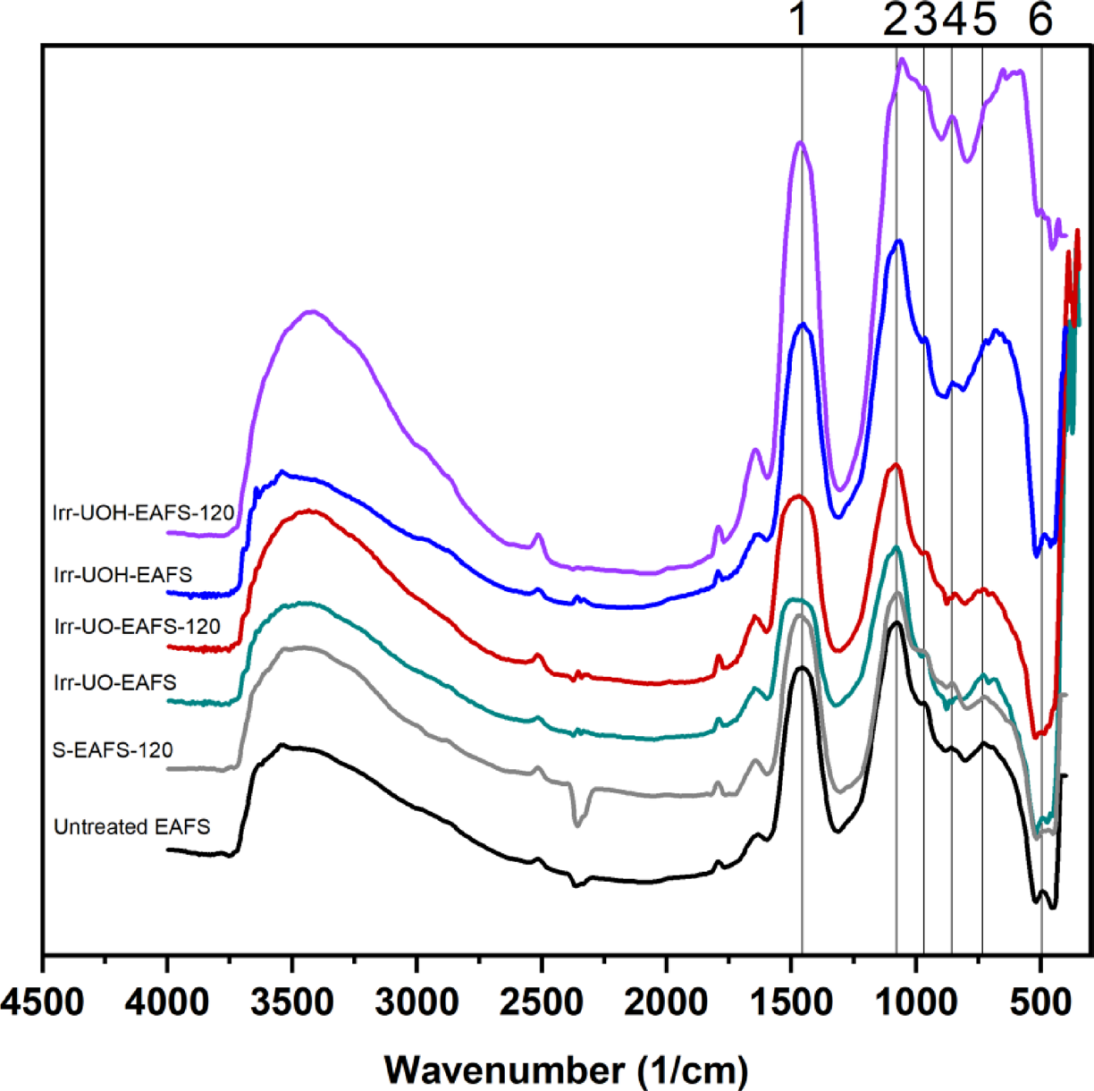
FTIR spectra of the electric arc furnace slag sample series.

Figure 7 presents the FTIR spectra of the series of electric arc furnace slag (EAFS) samples before and after HEPI and dissolution testing. As illustrated in Fig. 7, the left side denotes the untreated and high energy photon irradiated EAFS, whereas the right side shows those subjected to the dissolution test. The analysis concentrates solely on the fingerprint region, ranging from 1600 to 400 cm^− 1^, with the identified peaks labeled 1 through 6.

Upon detailed investigation of the untreated EAFS concerning the identified vibrational modes, peak #1 may be associated with the stretching units of Si-O bonds^65^. Similarly, peaks #2 and #3 may correspond to the formation of *T*-O-*T* vibrational units, where *T* represents Al or Si^66^. The peaks identified as #4 and #5 may be due to the stretching vibrations of Al-O and the bending units of *T*-O-*T* bonds, respectively, commonly found in the aluminosilicate structures^67^. Lastly, peak #6 is likely associated with the stretching units of Fe-O, potentially existing from iron-containing phases such as RO-phase or wustite^60^. The exposure of HEPI to untreated EAFS slightly affects the vibrational units of the Irr-UO-EAFS sample. At the same time, moderate alterations are observed in the Irr-UOH-EAFS powders. Irr-UO-EAFS exhibits similarities to untreated EAFS in terms of peak positions and intensities; however, Irr-UOH-EAFS shows a decline in both aspects. The increased total doses might modify the vibrational modes of EAFS.

Following the dissolution process, FTIR spectra exhibit variations across different samples. Applying the reference stirring technique to the untreated EAFS resulted in negligible changes in peak positions and their intensities. The limited element releases may be ascribed to the corresponding sample. Peak positions and intensities demonstrate changes after the dissolution test in the high energy photon irradiated samples. The peaks numbered 4 to 6 reveal differences from those found in the untreated EAFS sample. The alterations are more significant in the Irr-UOH-EAFS-120 sample, which underwent greater total doses of exposure. Another justification can be made based on the corresponding element releases presented in Fig. 5; higher element releases indicate more significant structural alterations. Consequently, it can be concluded that vibrational modes are unlikely to undergo significant changes before the dissolution test; however, their influence may be more pronounced during the dissolution of the samples.

### BET results

Table 3 displays the results of BET analysis to elucidate the variation in the specific surface area (SSA) of the untreated and high energy photon irradiated sample series. The untreated BM and EAFS values were obtained from our previous study^51^. In the case of BM, the SSA increases with variations in the applied HEPI, such that the highest total dose yields the greatest SSA. The SSA of the untreated EAFS is relatively greater than that of the untreated BM. Furthermore, the alteration by HEPI facilitates obtaining increased SSA, as shown by the observed values. This issue may be linked to the impact of the implemented HEPI techniques on the enhancement of SSA.

**Table 3:** Specific surface area of the related sample series.

Sample	SSA ( x10^3^ cm^2^/g)
Untreated BM	1.7
Irr-UO-BM	5.8
Irr-UOH-BM	8.9
Untreated EAFS	25.5
Irr-UO-EAFS	39.7
Irr-UOH-EAFS	44.8

### SEM analysis

**Fig. 8:**
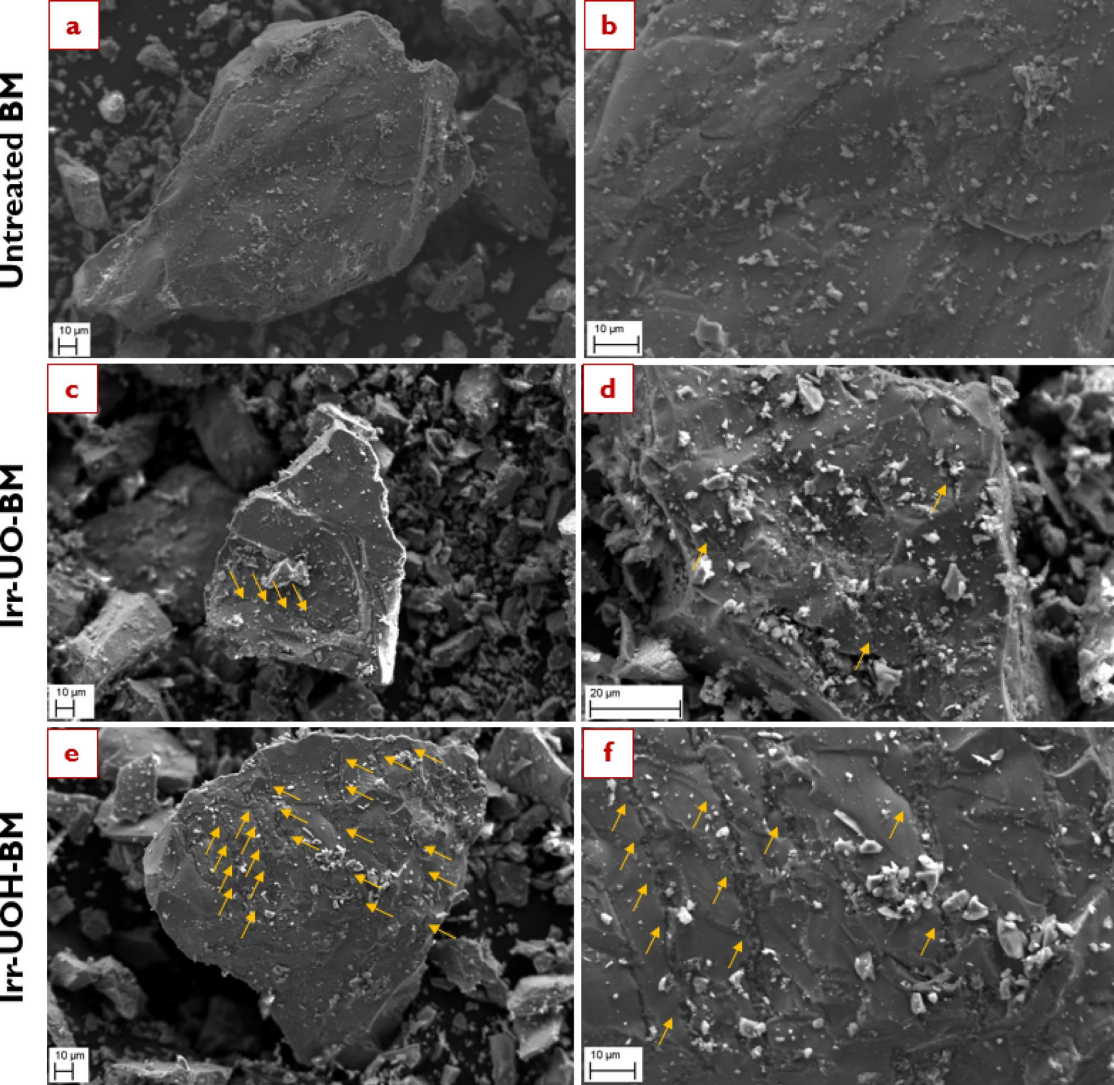
Surface morphology of the synthetic brownmillerite sample series before dissolution (Images **a**, **c**, and **e** were captured at 1000 X while Images–**b**, **d**, and **f** at 2500 X magnification).

The surface morphology of the synthetic brownmillerite (BM) was investigated using scanning electron microscopy (SEM). Images of the untreated and high energy photon irradiated samples were obtained at two magnifications, as shown in Fig. 8. For example, images *a* & *b* belong to the untreated BM sample captured at 1000 X and 2500 X, respectively. Following that, one may examine the surface morphology of the related sample series in detail. The untreated BM sample exhibits a smooth surface with an irregular form and sharp corners. Some literature investigations have also identified analogous surface morphology to that captured herein^68,69^. Specificly for Cs-137 irradiation (i.e., Irr-UO-BM) induces few micro-cracks on the surface, indicated by orange arrows in images *c* & *d*. A micro-crack line is particularly evident in image *c*, originating at the corner and extending to the opposite side. Similarly, other short micro-crack lines are depicted in image *d* (marked by the orange arrows). After detailed examination of images *e* & *f* (i.e., Irr-UOH-BM) related to a second HEPI (i.e., 10 MeV photons), it is obvious that both the number of micro-cracks and their widths increase. Image *f* provides enhanced comprehension by displaying multiple micro-crack lines in a parallel alignment. The findings suggest that HEPI procedures create micro-cracks on the sample’s surface, as there were significant differences between the untreated and high energy photon-irradiated sample series of BM. The increased total doses from 10 MeV photons impacted the sample’s surface far more significantly than Cs-137, forming multiple micro-crack lines. Hence, it can be said that the implemented HEPI treatments can influence the surface morphology already with low dose levels.

**Fig. 9:**
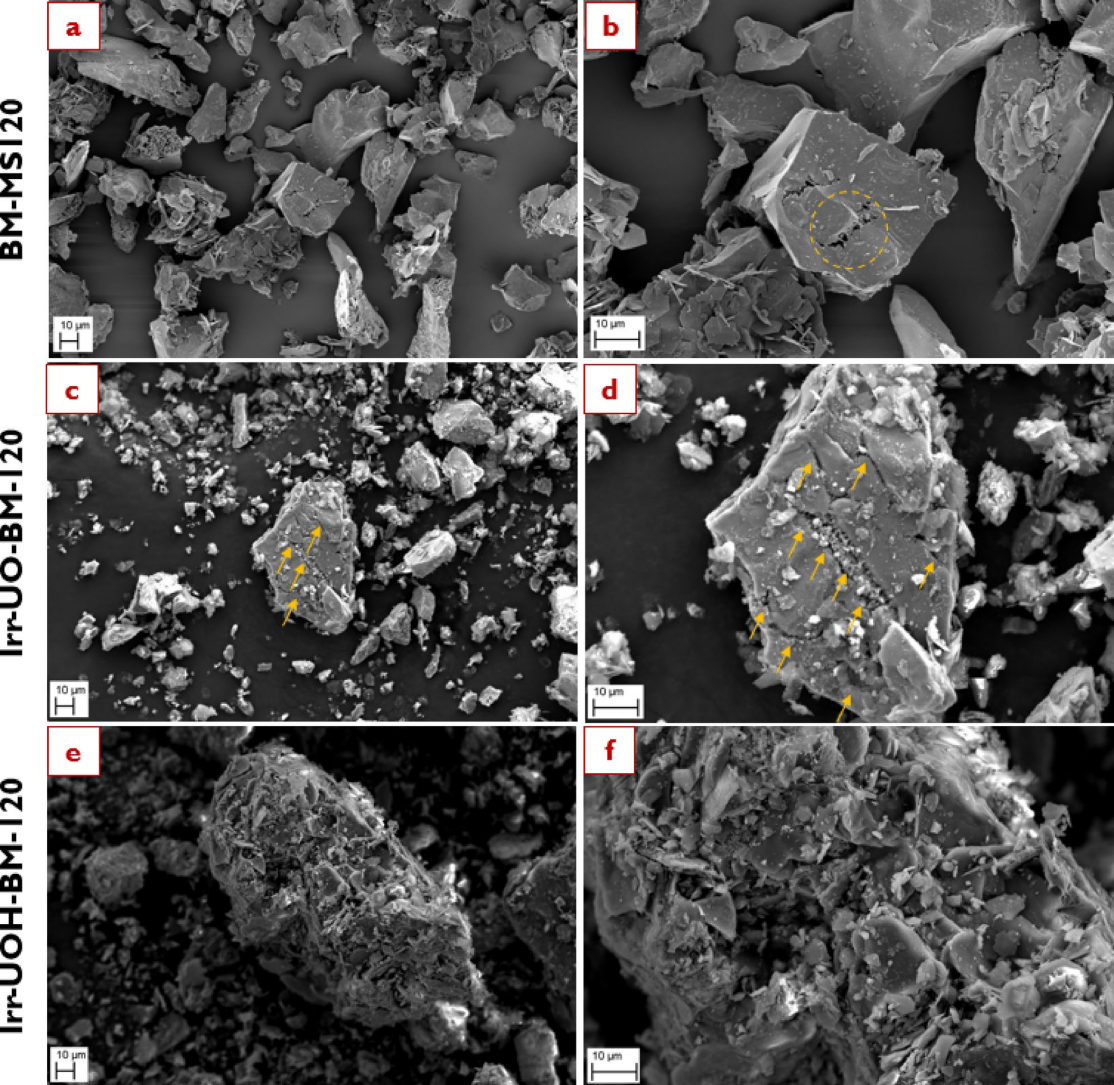
Surface morphology of the synthetic brownmillerite sample series after 120 min dissolution (Images **a**–**e** were captured at 1000 X while Images **b**–**f** at 2500 X magnification).

After understanding the surface morphology of the BM sample series prior to the dissolution process, Fig. 9 illustrates the surface morphologies subsequent to the dissolution test. The emphasis was directed towards the high energy photon irradiated sample series depicted in the figure, with BM-MS120, Irr-UO-BM-120 and Irr-UOH-BM-120 samples illustrated in images *a* & *b*, *c* & *d*, and *e & f* respectively. A detailed analysis reveals that the dissolution process in aqueous media resulted in substantial surface alteration in both samples relative to their high energy photon irradiated counterparts (see images *c* & *e* in Fig. 8). After the dissolution test, it can be seen that dissolution occurs largely from the micro-cracks which appear more porous and deep than before the dissolution test. Moreover, the number of observable micro-cracks in the Irr-UO-BM-120 sample increases, and their width expands. The orange arrows indicate the fractured areas in images *a* & *b*. Irr-UOH-BM-120 exhibits increased surface deterioration after its dissolution period, indicating high release of elements from the surface of particles. The dissolution process continued along the micro-crack lines in both scenarios, facilitating species release from the surface into the solution. The micro-cracks behaved as surface defects, increasing the specific surface area and ensuring greater contact points between liquid and solid. Although the crystal structure of the high energy photon irradiated samples remained unchanged (or might be under detection limit, e.g., 5–10%) post-exposure of high energy photons (Fig. 2), the results suggest that physical alterations on the surface (e.g., micro-cracks or surface fragments) may contribute to an increase in surface defects, thus enabling damage prior to the dissolution process. The elements may be rapidly liberated from these defect spots and released into the solution. Consequently, the quantities in the high energy photon irradiated samples were significantly increased compared to the reference (Fig. 4). In conclusion, the HEPI procedures compromised the surface morphology, induced micro-cracks, and augmented the specific surface area, resulting in improved dissolving properties and processing in aqueous environments.

**Fig. 10:**
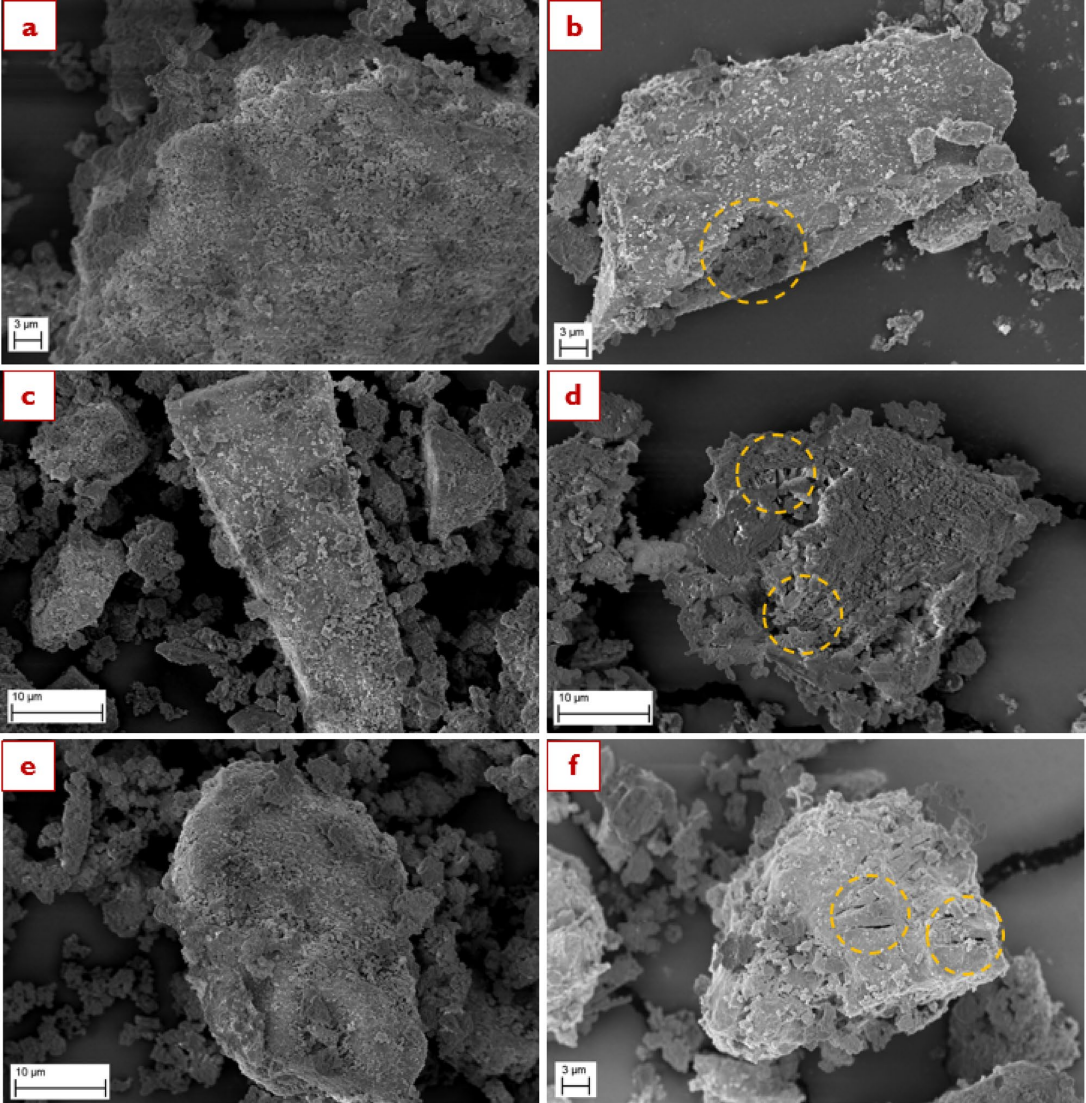
Surface morphology of the electric arc furnace slag sample series at 5000 X magnification (Images **a**, **c**, and **e** represent untreated, Cs-137-irradiated, and 10 MeV photon irradiated samples, respectively. Also, Images **b**, **d**, and **f** signify the corresponding samples after 120 min dissolution test, respectively).

Figure 10 exhibits the surface morphology of the EAFS samples series at 5000 X magnification. Images *a*, *c*, and *e* typify the untreated, Cs-137 irradiated, and 10 MeV photon irradiated samples before dissolution, respectively. In addition, images *b*, *d*, and *f* signify the corresponding samples following the 120 min dissolution test, respectively. According to the left-hand side images, the irregularly shaped particle with a complex surface morphology in Image *a* belongs to the untreated EAFS. The exposure to two different HEPI methods of EAFS, on the other hand, does not have a reasonable change in the surface morphology, such as the creation of microcracks, based on the Images *c* & *e*. Nevertheless, it might be suggested that nano-sized cracks or surface voids could be created due to HEPI if BET findings are considered (see Table 3). Because there are slight increments in the specific surface areas, depending on the doses applied via Cs-137 or 10 MeV photons. Even though distinctive alterations are not observed among the untreated and HEPI EAFS samples, the corresponding samples following the 120-minute dissolution test reveal some prominent differences. Image *b* shows the dissolved regions on EAFS particles marked with orange dashed line. With Cs-137 irradiation, more regions could be attained (see orange circled regions), proving the deterioration of the surface, which leads to increased element releases compared to the untreated form of EAFS. Moreover, multiple microcracks on the surface and related dissolved regions can be observed on samples that were treated with 10 MeV photon irradiation. This means a more pronounced effect of higher accumulated doses, paving the way for higher element releases. As a result of these observations, it can be said that surface morphology may seem unchanged following the EAFS’s HEPI – yet it is suggested to consider nano-sized cracks or surface voids. However, dissolved regions vary reasonably among the untreated and high energy photon irradiated EAFS samples following the dissolution test, supporting the potential impact of HEPI on the particle surface.

### XPS spectra

**Fig. 11:**
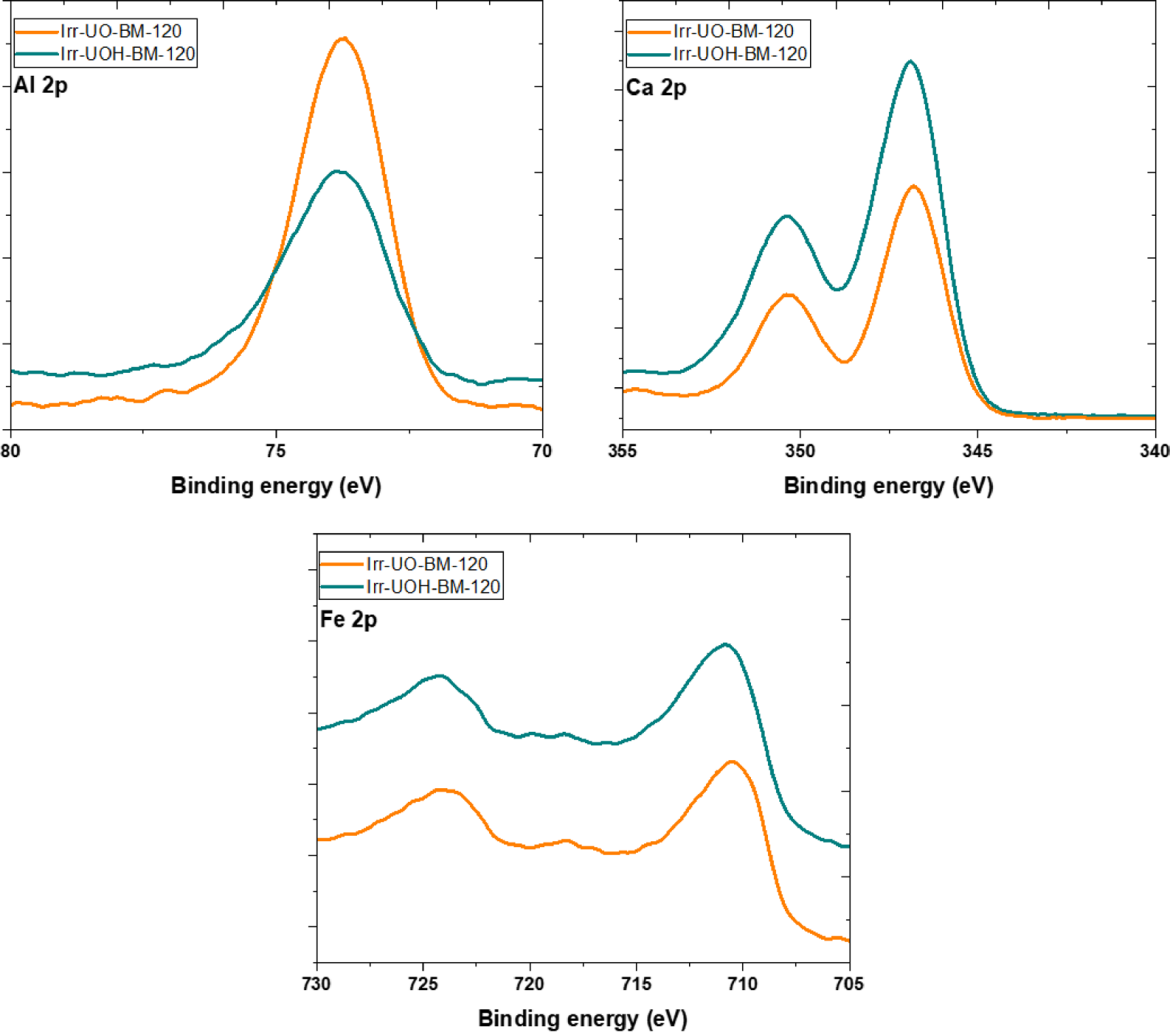
XPS spectra of the synthetic brownmillerite sample series.

XPS analysis was employed to ascertain the chemical state and surface chemistry of the high energy photonirradiated synthetic brownmillerite (BM) and electric arc furnace slag (EAFS) sample series subjected to a dissolution process for 120 min. Figure 11 illustrates the peak binding energy (*PBE*) of the elements found within the synthetic BM series. The *PBE* of Al 2p, valued at 74.4 eV, has been shifted by 0.7 eV for the Irr-UO-BM-120 sample and 0.5 eV for the Irr-UOH-BM-120 sample. This may pertain to surface modification resulting from the dissolution process. In the instance of Ca 2p exhibiting two distinct peaks, namely Ca 2p^3/2^ at 346.5 eV and Ca 2p^1/2^ at 351 eV, the *PBE*s have been adjusted by 0.3 and 0.4 eV for the former peak, and by 0.6 and 0.7 eV for the latter peak, respectively. Finally, the Fe 2p spectrum exhibits two distinct peaks: Fe 2p^1/2^ at 724 eV and Fe 2p^3/2^ at 710 eV. The samples have been displaced marginally (~ 0.5 eV) from their original placements. All findings indicate that *PBE* of these elements remained essentially unchanged during the dissolution process. The sole distinction between the two samples in each chemical state is that the UOH series has a greater *PBE* than UO, attributable to their varying dissolving properties.

**Fig. 12:**
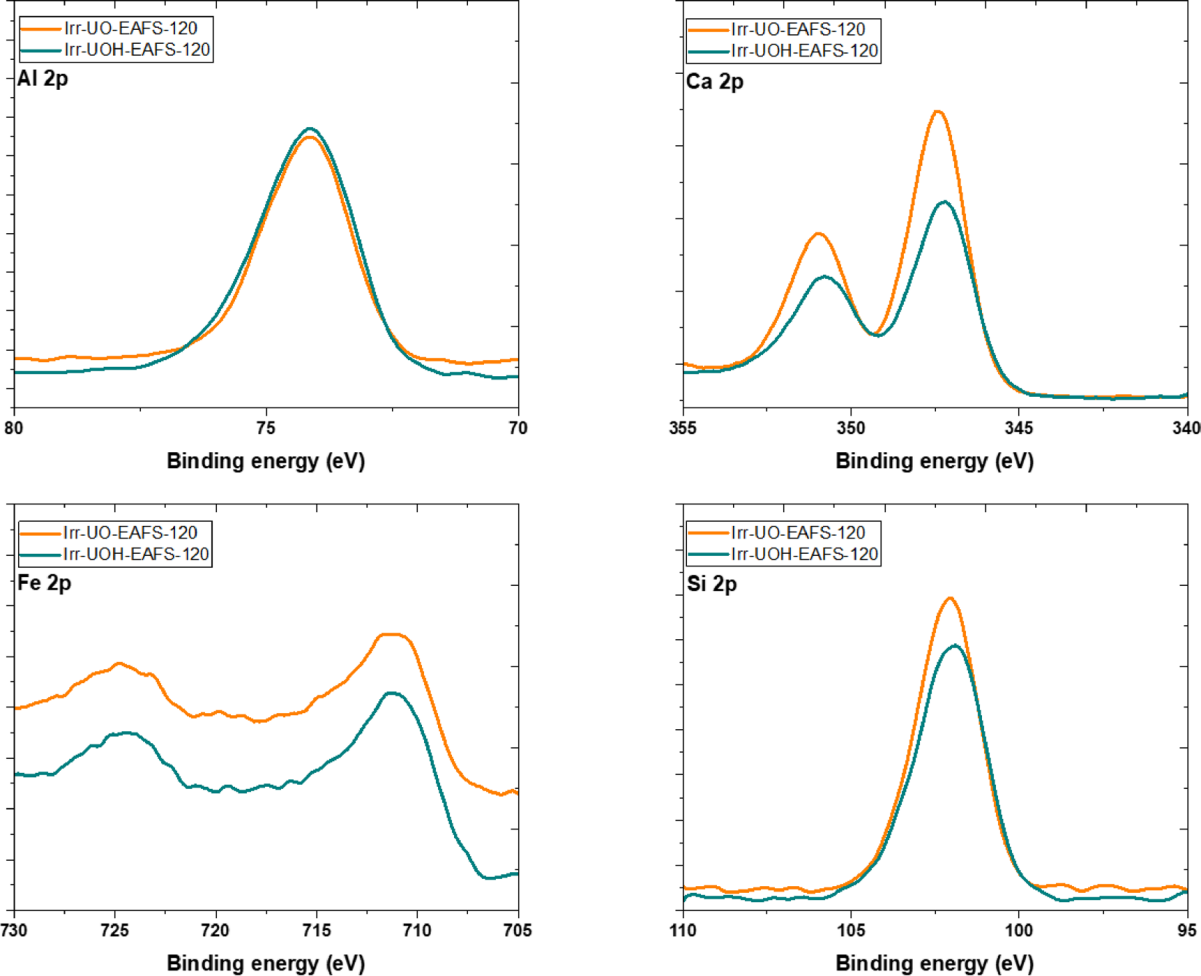
XPS spectra of the electric arc furnace slag sample series.

Figure 12 illustrates the *PBE* of the elements detected in the EAFS series, analogous to the XPS analysis of the synthetic BM series. According to the specified *PBE*s for the chemical states of Al 2p, Ca 2p, and Fe 2p, the samples Irr-UO-EAFS-120 and Irr-UOH-EAFS-120 exhibited minimal shifts, ~ 0.5 eV (Our previous publications^57,70^ can be found for the untreated EAFS spectra). Once more, the UOH series exhibited much greater *PBE*s in each element than the UO series, likely resulting from the distinct dissolving properties of their differing high energy photonirradiated forms. Another chemical state identified is Si 2p, typically located at 102 eV. The *PBE* has been altered by merely 0.2 eV in conjunction with the corresponding sample series. Consequently, it may be argued that the *PBE*s are comparable to each other.

**Fig. 13:**
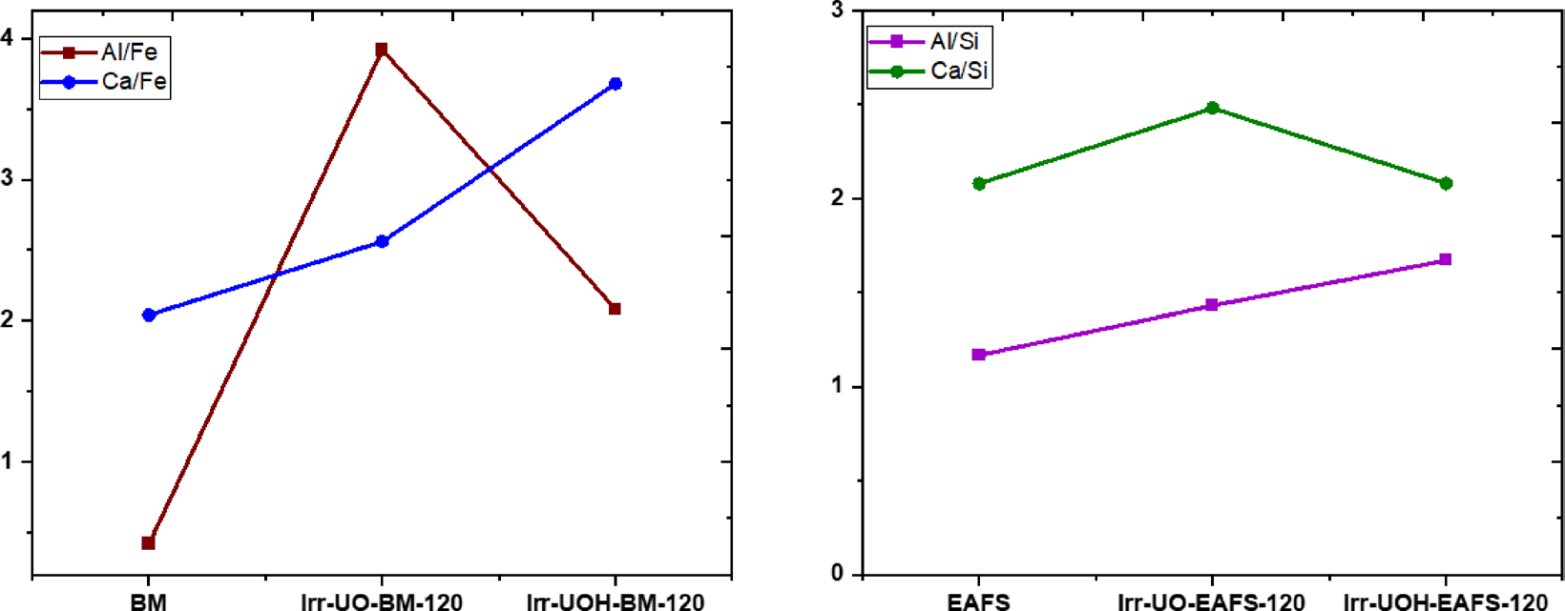
Surface chemistry of the synthetic brownmillerite (left) and electric arc furnace slag (right) sample series based on XPS analysis.

After examining the binding energies of the selected elements and corresponding samples, Fig. 13 illustrates the surface element ratios of Al/Fe & Ca/Fe and Al/Si & Ca/Si for the BM and EAFS series, respectively. The BM and EAFS data for the respective ratios in the figure were derived from their initial chemical compositions determined using Table 1 and Ref.^51^. In the BM series, the Al/Fe and Ca/Fe ratios are around 0.4 and 2.0, respectively; however, these ratios undergo substantial alterations following the dissolution period. The Al/Fe ratio rises to 3.9 after Cs-137 irradiation (UO) and dissolution test. This ratio also increases with the 10 Mev photon (UOH) series. These circumstances suggest that Al is prone to precipitate or adsorb on the surface due to the intricacies of the dissolving process. Similar to Al/Fe ratio changes, the Ca/Fe ratio increases after HEPI and dissolution tests. The UOH series exhibits the highest Ca/Fe ratio, indicating that the surface accumulation of Ca surpasses that of the UO series. Consequently, it can be asserted that both elements accumulate on the surface during the dissolution process, and these accumulations undoubtedly influence the ultimate dissolution concentrations of the elements, as previously illustrated in Fig. 4.

Furthermore, one may adhere to the element ratios, namely Al/Si and Ca/Si, for EAFS, as depicted in Fig. 13. The Al/Si and Ca/Si ratios are around 1.17 and 2.08, respectively. Dissolving the high energy photon irradiated EAFS samples in aqueous solutions substantially alters the element ratios, increasing both. The ratio increase likely indicates species accumulation on the surface, as no additional silicon has been released into the solution. The accumulation of aluminum is significantly more pronounced in the UO series. Conversely, Ca tends to accumulate when the UOH series is pertinent, analogous to the behavior noted in the BM sample series. Nonetheless, both components tend to accumulate on the surface through precipitation or adsorption, potentially influencing the resultant dissolution concentrations of the solution.

## Discussion

The synthetic brownmillerite (BM) and electric arc furnace slag (EAFS) sample series initially underwent HEPI procedures utilizing Cs-137 (UO) and 10 MeV photons from linear accelerator (UOH) sources. The untreated and high energy photon irradiated forms were subsequently dissolved in an aqueous environment to examine the element releases.

Brownmillerite (Ca_2_AlFe_2_O_5_) crystallizes in an orthorhombic structure (Ibm2) with alternating layers of tetrahedrally and octahedrally coordinated Fe/Al units corner-sharing along the b axis, which facilitates ion mobility and affect radiation interaction behaviors^71,72^. Fe^3+^is mostly hosted in octahedral sites, whereas Al^3+^ occurs preferentially in tetrahedral sites. Calcium atomic sites are located between tetrahedral and octahedral layers, representing irregular polyhedron made of eight oxygen atoms^73^. In the case of BM samples, exposure of the untreated powders to HEPI did not result in any observable structural changes, as determined by XRD and FTIR analyses. The peaks identified in the XRD patterns and the vibrational modes observed in the FTIR spectra were broadly analogous across the sample series. Notwithstanding these facts, the element releases increased relative to the reference when applying HEPIs. Given the comparable structures of the untreated and high energy photon irradiated sample series, attention may be directed towards the surface morphology analysed by SEM, as the surface significantly influences the dissolution properties of minerals^74,75^. Figure 8 demonstrated surface morphology alterations before and after HEPI. Subjecting untreated BM powders to an increased total dose via 10 MeV photons from linear accelerator led to the formation of micro-crack lines. The alteration in surface morphology can also be corroborated by observing specific surface area values (refer to Table 3).

On the other hand, no observable structural alterations were detected in the EAFS sample series by XRD and FTIR analyses after HEPI procedure, similar to the BM sample series. This situation might be associated with the fact that the amount or volume of these alterations might be lower than what can be detected with XRD and FTIR since there were no changes in their corresponding data. In addition, unchanged structures were also reported by some studies focusing on the effect of gamma irradiation, as well^76^. It is important to note that the element releases increased when the high energy photon irradiated sample series were dissolved in aqueous conditions, as observed in the case of BM. SEM analysis of EAFS (refer to Fig. 10, specifically images *a*, *c*, and *e*) did not reveal significant changes in surface morphology after HEPI procedures. However, specific surface areas exhibited an increasing trend corresponding to higher energy exposure, with SSA for 10 MeV photons surpassing that for Cs-137. The absence of observed changes in the surface morphology of EAFS may be attributed to the structural complexity characterized by the coexistence of multiple phases. Therefore, it can be concluded that neither HEPI procedure have the potential to damage the crystal structure of the samples, although they may alter the surface morphology to a certain degree.

From these findings, the authors canalized through speculating the potential reasons for the related outcomes. In the literature, some studies indicated that gamma irradiation can lead to local temperature increases in the material’s surface, which is influenced by various factors such as the applied source, total dose, duration, and distance parameters^77–79^. On the other hand, it has been suggested that the increase in the temperature and the exposure time to gamma irradiation could induce local thermal stresses on the material’s surface, potentially paving the way for micro-crack formations on the surface^80,81^. Such thermally driven stress gradients may also interact with pre-existing structural heterogeneities or voids, amplifying crack nucleation under prolonged irradiation. In the occurrences of thermal stresses, it might be too complex to quantify the temperature differences or changes on the surface (at least, no observation was made in this study). This is because of the facts that the interaction between matter and ray becomes critical in addition to considering the specific heat capacity of the material itself, as well as high energy photon radiation absorption characteristics of the material of interest (for instance, elements with high atomic number would absorb more HEPI). In addition to thermal effects, high-energy photons—particularly in the MeV range—can interact with matter through the photoelectric effect, Compton scattering, and pair production, which generate energetic secondary electrons. These electrons can cause local ionization, bond scission, and creation of point defects (e.g., oxygen vacancies), particularly at or near the surface. One may speculate that, by heavy atoms included within the material, high energy photon might be absorbed to a certain degree, contingent upon the concentration of the heavy atoms within BM and EAFS^82^. When high energy photon interact with matter, three established mechanisms are observed: the photoelectric effect, Compton scattering, and pair production^19^. Due to these mechanisms, the energy absorption build-up factor becomes a focal point of interest. That is, the deposited energy within the material might enhance the overall impact of gamma irradiation^18^. Using a 10 MeV photon source might result in more significant temperature differences, which might induce more thermal stress on the surface, particularly concerning the duration of exposure (i.e. 16 h) and total doses (i.e., 52 kGy). Temperature changes do not reasonably alter the structure of the material systems, as these changes are inadequate to cause amorphization or modify the crystal phases. However, it might be reasonable that the surface could be affected by the formation of micro-cracks resulting from thermal shocks and stresses, which can enhance the specific surface area and finally improve the dissolution properties of the powders. In parallel, the radiation-induced formation of surface defects and local coordination disruptions can further increase the material’s chemical reactivity, particularly by facilitating hydroxylation and surface hydration reactions upon exposure to water. These defect-assisted processes may thus work in tandem with microcracking to promote dissolution, even in the absence of detectable bulk structural damage.

## Conclusions

This study investigated the impact of high energy photon irradiation at two distinct dosages, such as Cs-137 isotope (0.662 MeV, 250 Gy) and medical linear accelerator (10 MeV, 52 kGy), on synthetic brownmillerite and electric arc furnace slag. This work also analyzed the dissolution properties of the irradiated powders in aqueous environments. The data indicate that the high energy photon irradiations did not induce structural or vibrational alterations in either sample as analyzed by XRD and FTIR. Notwithstanding this, the element releases, namely Al and Ca, increased when the high-energy photon irradiated powders were dissolved in water, in contrast to the reference samples. The samples subjected to elevated doses from the 10 MeV photon source had the greatest releases of Al and Ca, achieving up to 20% and 10% extent of dissolution, respectively. In contrast, the reference produced only half of those percentages. Despite no apparent change in crystallographic structure, the increasing dissolution could be related to changes on the surface of particles resulting from high-energy photon irradiation. Microstructural analyses conducted by scanning electron microscopy detected the formation of numerous micro-cracks, particularly in the synthetic brownmillerite, which was more pronounced in the 10 MeV photon irradiated sample. The dissolution process appeared to occur along the micro-crack lines, as evidenced by the increasing width and depths of the cracks after the dissolution tests. It has been assumed that the thermal stresses on the surface, resulting from temperature differences, could be the underlying cause of the surface alterations. The authors noted that synthetic brownmillerite and electric arc furnace slag may not experience extensive structural modifications due to high energy photon irradiation; nonetheless, both can experience significant changes in particle stiffness, leading to micro-crack development. The specific surface areas of both samples, synthetic brownmillerite, and electric arc furnace slag, increased by the rising total doses from the sources. The results of this work provide relevant considerations when planning the utilization of industrial mineral wastes in applications in which they could be exposed to high-energy photon irradiation.

## Data Availability

The datasets used and/or analysed during the current study available from the corresponding author on reasonable request.
